# Prevalence and Characteristics of Autism Spectrum Disorder Among Children Aged 8 Years — Autism and Developmental Disabilities Monitoring Network, 11 Sites, United States, 2012

**DOI:** 10.15585/mmwr.ss6513a1

**Published:** 2018-11-16

**Authors:** Deborah L. Christensen, Kim Van Naarden Braun, Jon Baio, Deborah Bilder, Jane Charles, John N. Constantino, Julie Daniels, Maureen S. Durkin, Robert T. Fitzgerald, Margaret Kurzius-Spencer, Li-Ching Lee, Sydney Pettygrove, Cordelia Robinson, Eldon Schulz, Chris Wells, Martha S. Wingate, Walter Zahorodny, Marshalyn Yeargin-Allsopp

**Affiliations:** 1 Division of Congenital and Developmental Disorders, National Center on Birth Defects and Developmental Disabilities, CDC; 2 University of Utah, Salt Lake City; 3 Medical University of South Carolina, Charleston; 4 Washington University in St. Louis, Missouri; 5 University of North Carolina, Chapel Hill; 6 University of Wisconsin–Madison; 7 University of Arizona, Tucson; 8 Johns Hopkins University; 9 University of Colorado at Denver and Health Sciences Center; 10 University of Arkansas for Medical Sciences, Little Rock; 11 Colorado Department of Public Health and Environment, Denver; 12 University of Alabama at Birmingham; 13 Rutgers University–New Jersey Medical School, Newark

## Abstract

**Problem/Condition:**

Autism spectrum disorder (ASD).

**Period Covered:**

2012.

**Description of System:**

The Autism and Developmental Disabilities Monitoring (ADDM) Network is an active surveillance system that provides estimates of the prevalence and characteristics of ASD among children aged 8 years whose parents or guardians reside in 11 ADDM Network sites in the United States (Arkansas, Arizona, Colorado, Georgia, Maryland, Missouri, New Jersey, North Carolina, South Carolina, Utah, and Wisconsin). Surveillance to determine ASD case status is conducted in two phases. The first phase consists of screening and abstracting comprehensive evaluations performed by professional service providers in the community. Data sources identified for record review are categorized as either 1) education source type, including developmental evaluations to determine eligibility for special education services or 2) health care source type, including diagnostic and developmental evaluations. The second phase involves the review of all abstracted evaluations by trained clinicians to determine ASD surveillance case status. A child meets the surveillance case definition for ASD if one or more comprehensive evaluations of that child completed by a qualified professional describes behaviors that are consistent with the *Diagnostic and Statistical Manual of Mental Disorders, Fourth Edition, Text Revision* diagnostic criteria for any of the following conditions: autistic disorder, pervasive developmental disorder–not otherwise specified (including atypical autism), or Asperger disorder. This report provides ASD prevalence estimates for children aged 8 years living in catchment areas of the ADDM Network sites in 2012, overall and stratified by sex, race/ethnicity, and the type of source records (education and health records versus health records only). In addition, this report describes the proportion of children with ASD with a score consistent with intellectual disability on a standardized intellectual ability test, the age at which the earliest known comprehensive evaluation was performed, the proportion of children with a previous ASD diagnosis, the specific type of ASD diagnosis, and any special education eligibility classification.

**Results:**

For 2012, the combined estimated prevalence of ASD among the 11 ADDM Network sites was 14.5 per 1,000 (one in 69) children aged 8 years. Estimated prevalence was significantly higher among boys aged 8 years (23.4 per 1,000) than among girls aged 8 years (5.2 per 1,000). Estimated ASD prevalence was significantly higher among non-Hispanic white children aged 8 years (15.3 per 1,000) compared with non-Hispanic black children (13.1 per 1,000), and Hispanic (10.2 per 1,000) children aged 8 years. Estimated prevalence varied widely among the 11 ADDM Network sites, ranging from 8.2 per 1,000 children aged 8 years (in the area of the Maryland site where only health care records were reviewed) to 24.6 per 1,000 children aged 8 years (in New Jersey, where both education and health care records were reviewed). Estimated prevalence was higher in surveillance sites where education records and health records were reviewed compared with sites where health records only were reviewed (17.1 per 1,000 and 10.4 per 1,000 children aged 8 years, respectively; p<0.05). Among children identified with ASD by the ADDM Network, 82% had a previous ASD diagnosis or educational classification; this did not vary by sex or between non-Hispanic white and non-Hispanic black children. A lower percentage of Hispanic children (78%) had a previous ASD diagnosis or classification compared with non-Hispanic white children (82%) and with non-Hispanic black children (84%). The median age at earliest known comprehensive evaluation was 40 months, and 43% of children had received an earliest known comprehensive evaluation by age 36 months. The percentage of children with an earliest known comprehensive evaluation by age 36 months was similar for boys and girls, but was higher for non-Hispanic white children (45%) compared with non-Hispanic black children (40%) and Hispanic children (39%).

**Interpretation:**

Overall estimated ASD prevalence was 14.5 per 1,000 children aged 8 years in the ADDM Network sites in 2012. The higher estimated prevalence among sites that reviewed both education and health records suggests the role of special education systems in providing comprehensive evaluations and services to children with developmental disabilities. Disparities by race/ethnicity in estimated ASD prevalence, particularly for Hispanic children, as well as disparities in the age of earliest comprehensive evaluation and presence of a previous ASD diagnosis or classification, suggest that access to treatment and services might be lacking or delayed for some children.

**Public Health Action:**

The ADDM Network will continue to monitor the prevalence and characteristics of ASD among children aged 8 years living in selected sites across the United States. Recommendations from the ADDM Network include enhancing strategies to 1) lower the age of first evaluation of ASD by community providers in accordance with the *Healthy People 2020* goal that children with ASD are evaluated by age 36 months and begin receiving community-based support and services by age 48 months; 2) reduce disparities by race/ethnicity in identified ASD prevalence, the age of first comprehensive evaluation, and presence of a previous ASD diagnosis or classification; and 3) assess the effect on ASD prevalence of the revised ASD diagnostic criteria published in the *Diagnostic and Statistical Manual of Mental Disorders, Fifth Edition*.

## Introduction

Autism spectrum disorder (ASD) is a developmental disability characterized by social and communication impairments and by restricted interests and repetitive behaviors (*1*). The first studies of the prevalence of autism were published in the 1960s and 1970s, when autism was thought to be a very severe condition, usually accompanied by intellectual disability (*2*). These studies reported the prevalence to be approximately four to five cases per 10,000 children. Autism was first distinguished as a unique clinical diagnosis by the American Psychiatric Association with the publication in 1980 of the third edition of the *Diagnostic and Statistical Manual of Mental Disorders* (DSM-III) (*3*), which provided diagnostic criteria for infantile autism and pervasive developmental disorder. Since that time, autism has become recognized as a spectrum of behavioral characteristics, which results in varying degrees of functional limitations. In 1994, the *Diagnostic and Statistical Manual of Mental Disorders, Fourth Edition* (DSM-IV) introduced revised diagnostic criteria and five subtypes of autism, including autistic disorder, Asperger disorder, pervasive developmental disorder–not otherwise specified (PDD-NOS), childhood disintegrative disorder, and Rett’s disorder (*4*). The first three subtypes comprise autism spectrum disorder (ASD), whereas the latter two conditions belong to the wider category of pervasive developmental disorders. The fifth edition of DSM, which was published in 2013 (*5*), redefined ASD as a single disorder, along with other changes in the diagnostic classification of ASD. For this report, the evaluations contained in children’s records were conducted no later than 2012, and therefore DSM-IV-TR diagnostic criteria were used in the ASD surveillance case definition to estimate ASD prevalence and characteristics.

Substantial increases in the estimated prevalence of ASD in the United States have been reported since the 1990s. Two studies conducted in the late 1980s that used DSM-III screening and diagnostic criteria for pervasive developmental disorder estimated prevalence as 3.3 cases per 10,000 children aged 3–18 years (*6*) and 3.6 cases per 10,000 children aged 8–12 years (*7*). Since then, increases in estimated ASD prevalence have been measured, using data from special education and other administrative records (*8*–*10*), national surveys (*11*–*14*), and active public health surveillance conducted through CDC’s Metropolitan Atlanta Developmental Disabilities Surveillance Program (MADDSP) and its extended surveillance network, the Autism and Developmental Disabilities Monitoring (ADDM) Network. MADDSP first estimated ASD prevalence among children aged 3–10 years in 1996 to be 3.4 per 1,000 children aged 3–10 years (*15*). Subsequently, the larger ADDM Network estimated prevalence across multiple U.S. sites every 2 years during 2000–2010. The most recent prevalence estimate from the ADDM Network for children aged 8 years was 14.7 per 1,000 children in 2010 (*16*), compared with 11.3 in 2008 (*17*), 9.0 in 2006 (*18*), 6.6 in 2002 (*19*), and 6.7 in 2000 (*20*).

The American Academy of Pediatrics recommends that pediatric health care providers administer two ASD screenings, at ages 18 and 24 months, using a valid and reliable screening tool (*21*). Children whose screening results are concerning should subsequently receive a comprehensive developmental evaluation from a general or developmental pediatrician, child neurologist, child psychiatrist, or child psychologist, which can be obtained privately or through the Part C (ages 0–<3 years) or Part B (ages 3–21 years) programs of the Individuals with Disabilities Education Act (http://idea.ed.gov/explore/home) supported by each state. To support and measure progress in early identification, the *Healthy People 2020* initiative includes a goal to increase the percentage of children with ASD who receive their first comprehensive evaluation by age 36 months by 10%, from the baseline of 42.7% in 2006 to the goal of 47.0% in 2020 (*22*). ADDM Network ASD surveillance data for children aged 8 years are used to evaluate progress toward this goal.

This report describes estimated ASD prevalence and characteristics among children aged 8 years in the ADDM Network in 2012. This includes 1) total estimated ASD prevalence as well as prevalence by surveillance site, sex, and race/ethnicity and 2) characteristics of children with ASD, including presence of intellectual disability, age at earliest known comprehensive evaluation, presence of a previous ASD diagnosis or educational classification, age at previous ASD diagnosis and diagnosis subtype, and special education eligibility classification. The intended audience for this report includes health care providers, early intervention service providers, therapists, school psychologists, educators, researchers, policymakers, and program administrators seeking to understand and provide for the needs of persons with ASD and their families. These data can be used to help plan for service needs, stimulate research into etiology and risk factors, and initiate and implement policies that promote optimal outcomes in health care and education.

## Methods

### ADDM Network Sites

The ADDM Network is an active surveillance system that provides estimates of the prevalence and characteristics of ASD among children aged 8 years whose parents or guardians reside within 11 ADDM sites in the United States (selected counties or parts of counties in Arkansas, Arizona, Colorado, Georgia, Maryland, Missouri, New Jersey, North Carolina, South Carolina, Utah, and Wisconsin). The ADDM Network uses multisite, multiple-source surveillance based on a review of behavioral descriptions and ASD diagnoses documented in comprehensive developmental evaluations present in children’s health and education records, using a model developed by CDC’s MADDSP (*15*,*23*). The ADDM Network sites were selected through a competitive process, with preference for a diverse population in terms of race/ethnicity. Each ADDM site functions as a public health authority under the Health Insurance Portability and Accountability Act of 1996 Privacy Rule and meets applicable local Institutional Review Board, privacy, and confidentiality requirements under 45 CFR 46 (*24*).

### Case Ascertainment

Children eligible for case ascertainment were born in 2004, and their parents or guardians lived in site-specific ADDM Network surveillance catchment areas at some time during 2012. At each site, surveillance data were linked to the state’s 2004 birth certificate records to obtain data on race/ethnicity and other demographic characteristics. If a birth certificate match was not made, the child was assumed to have been born outside the state. No clinical examinations of children were performed by ADDM Network staff.

ADDM Network investigators use a two-phase surveillance approach to ascertain potential ASD cases. The first phase involves screening and abstracting records from multiple data sources in the community, including special education programs and health care providers who evaluate and treat children with developmental disabilities. Agreements to access records are made at the institutional level in the form of contracts, memoranda, or other formal agreements. In the second phase, all abstracted evaluations are compiled and reviewed by clinicians with specialized training in the evaluation and diagnosis of ASD, including physicians, psychologists, and speech/language pathologists. These clinician reviewers follow the ADDM surveillance protocol to determine ASD case status and to maintain reliability.

Data sources identified for record review are categorized as either 1) education source type, including developmental evaluations to determine eligibility for special education services or 2) health care source type, including diagnostic and developmental evaluations. All ADDM Network sites have agreements in place to access records at health care sources. For the 2012 surveillance year, six sites (Arizona, Georgia, New Jersey, North Carolina, South Carolina, and Utah) also reviewed education records in all or most of the surveillance area. In the Maryland site, education records were reviewed in one of the six participating counties, and in the Colorado site, education records were reviewed for some of the public school districts in one of the seven counties in the surveillance area. For these two surveillance sites, only health care source type records were reviewed in the remaining counties. Three sites (Arkansas, Missouri, and Wisconsin) reviewed records only at health care sources.

In the first phase of surveillance, ADDM Network sites identify source records to review on the basis of a child’s year of birth and either 1) eligibility classifications in special education or 2) *International Classification of Diseases, Ninth Revision, Clinical Modification* (ICD-9-CM) billing codes for select childhood disabilities or conditions. Children’s records are screened to confirm year of birth and whether the parent or guardian of the child lived in the surveillance area at any time during the surveillance year. For children meeting age and residency requirements, the source files are screened for specific behavioral or diagnostic descriptions defined by ADDM as triggers for abstraction. These triggers include a documented ASD classification (either a diagnosis of ASD or a special education placement category of ASD) and/or descriptions of behaviors consistent with an ASD diagnosis (e.g., limited or no interaction with other children or prefers objects over persons). If abstraction triggers are found, available information from birth through the current surveillance year is abstracted, including: 1) information on demographic characteristics; 2) other medical conditions; 3) evaluation dates and verbatim descriptions of behaviors consistent with ASD from comprehensive developmental evaluations by community professionals; 4) community professional type and degree (e.g., MD [neurologist, psychiatrist, or developmental pediatrician], PhD [psychologist], or EdS [education specialist]); 5) developmental history, including statements about parental or professional concerns that the child's development was atypical; 6) special education service category; 7) scores from intelligence quotient (IQ), adaptive, and autism diagnostic tests; and 8) evaluation conclusions. The most recent eligibility classification for receiving special education services (e.g., autism or learning disability) is collected from special education records. For all abstracted evaluations, information from multiple sources is combined into one composite summary record for each child.

In the second phase of surveillance, referred to as “clinician review,” the abstracted composite evaluation records are deidentified and reviewed systematically by clinicians who have undergone standardized training to determine ASD case status using a coding scheme based on the DSM-IV-TR (*1*) criteria for ASD. A child meets the surveillance case definition for ASD if behaviors described in the composite record are consistent with the DSM-IV-TR diagnostic criteria for any of the following conditions: autistic disorder, PDD-NOS (including atypical autism), or Asperger disorder. ASD surveillance case criteria were based on DSM-IV-TR because surveillance was conducted using records generated before or during 2012, prior to publication of new diagnostic criteria in the *Diagnostic and Statistical Manual of Mental Disorders, Fifth Edition* (DSM-5) (*5*). For the majority of children, one clinician reviews the composite record. If a child meets the surveillance case criteria, but the reviewer is uncertain whether ASD is the most appropriate classification, a second, independent review is done. Following the second review, the two reviewers meet and come to a consensus on the child’s case status.

### Descriptive Characteristics

The diagnostic summaries from each evaluation record were abstracted for each child, including age at and subtype of any previous ASD diagnoses. Children were considered to have a previously documented ASD classification if the child received a DSM-IV-TR diagnosis of autistic disorder, Asperger disorder, PDD-NOS, or ASD-NOS, or if ASD was documented by an ICD-9-CM billing code at any time from birth through the year when the child reached age 8 years, or if the child received special education services under an autism eligibility during the surveillance year. Information was collected on children’s functional abilities, including scores on standardized tests of intellectual ability. The most recently recorded scores from tests of a child’s intellectual ability were used to categorize the child in the intellectual disability range if the intelligence quotient (IQ) score was ≤70, in the borderline range if the score was 71–85, and in the average to above-average range if the score was >85. The child’s age at the earliest comprehensive evaluation was documented and is reported as the median age at the earliest comprehensive evaluation in months and as the percentage of children with an earliest known comprehensive evaluation performed by age 36 months. Information also was recorded about the age at which developmental concerns were documented in the records. Analyses of the age at earliest known comprehensive evaluation and the age at which developmental concerns first were documented were restricted to children who were born in the state in which they resided at age 8 years. This restriction was imposed to reduce bias that might have resulted from the unavailability of evaluations performed early in life when the child was residing in a state other than the state in which the ADDM Network site was located.

### Quality Assurance

All ADDM Network sites follow the same quality assurance protocol. In the first phase of case ascertainment, screening and abstraction of source records are monitored for accuracy by means of a 10% random sample of records to check the accuracy of data collection as well as the appropriate selection of the record for abstraction. Initial interrater reliability among ASD clinician reviewers was established to a minimum standard of 90% agreement on their decision about whether the child meets the ASD surveillance case definition defined in the ADDM study protocol prior to the beginning of the second phase of case ascertainment. Subsequently, interrater reliability for clinician reviewers is monitored on an ongoing basis using a blinded, random 10% sample of abstracted records that are scored independently by two reviewers. The final interrater agreement for determining surveillance ASD case status (ASD case versus not ASD case) was 90.4% when reliability samples from all ADDM Network sites were combined (ĸ = 0.8).

### Analytic Methods

The prevalence estimate of ASD among children in the ADDM Network was calculated as the number of children aged 8 years who met the surveillance ASD case definition across the 11 ADDM Network sites in 2012 divided by the number of children aged 8 years residing in the counties comprising the 11 surveillance sites. Population denominators used were obtained from CDC’s National Center for Health Statistics (NCHS) vintage 2014 postcensal bridged-race population estimates for 2012 (http://www.cdc.gov/nchs). In the Arizona site, only part of a county was included in the surveillance catchment area. Therefore, the number of children in this county who lived within the surveillance area was estimated in order to obtain the appropriate denominator. This was done by obtaining the third-grade school enrollment counts for the years 2012–2013 for the public school districts included in the surveillance area from the National Center for Education Statistics (https://nces.ed.gov). The number of third-grade students enrolled in the public school districts included in the surveillance area was divided by the number of third-grade students enrolled in all of the public school districts in the county to obtain the proportion of students enrolled in participating school districts. This proportion was then applied to the NCHS vintage 2014 postcensal bridged-race population estimate for each county in 2012 to obtain the relevant denominator. The bridged-race categories used in this report include non-Hispanic white, non-Hispanic black, Hispanic, American Indian/Alaska Native, and Asian/Pacific Islander. Data from all ADDM sites were pooled to produce combined ASD prevalence estimates. Prevalence estimates were stratified by surveillance site, sex, and race/ethnicity (i.e., non-Hispanic white, non-Hispanic black, and Hispanic). Other race/ethnicity groups were represented by too few children to generate stable estimates of ASD prevalence at all surveillance sites. The race/ethnicity of each child whose records were abstracted was determined from information contained in source records or, if not found in the source records, from birth certificates (when available). Hispanic refers to all children who are of Hispanic ethnicity, regardless of race. Overall prevalence estimates included all children identified with ASD regardless of sex, race/ethnicity, or intellectual ability and therefore were not limited to children with available data on these characteristics.

Statistical tests were used and confidence interval (CI) estimates were calculated following the assumption that the observed counts of ASD surveillance cases were drawn from an underlying Poisson sampling distribution. Pearson chi-square tests and prevalence ratios (PR) were used to examine the association between ASD prevalence estimates and characteristics of children with ASD by surveillance site, record source type, sex, and race/ethnicity. Exact tests were used when the number of children was fewer than five. The nonparametric median test was used to determine differences in median age at first evaluation for ASD and earliest known ASD diagnosis by sex and race/ethnicity. Statistical significance was set at p<0.05. All analyses were performed by using SAS statistical software (SAS Institute, Cary, North Carolina).

### Evaluation Methods

Some children who were identified for screening could not be included in the ADDM Network ASD case determination process because some or all of the education and health records could not be found for review. Therefore, an analysis was performed to determine the potential effect of these missing records on ASD prevalence estimates. All children initially identified for screening were first stratified by two factors closely associated with final case status: information source (health source type only, education source type only, or both source types) and the presence or absence of either an autism special education eligibility or an ICD-9-CM code for ASD, collectively forming six strata. The potential number of cases that might have been identified if missing records had been included was estimated by assuming that within each of these six strata, the proportion of children with ASD in each stratum (with and without missing records) would be similar. Subsequently, the proportion of children meeting the ASD surveillance case definition was applied to the number of children with missing records in the same stratum to estimate the number of missed cases and the corresponding increase in prevalence. Estimates from all six strata were added to produce the total for each site. The analysis of the potential effect of missing records was performed for evaluation purposes, and the prevalence estimates presented in this report do not reflect this adjustment.

### Comparison of Surveillance Sites between 2010 and 2012

For eight sites (Arizona, Colorado, Georgia, Missouri, New Jersey, North Carolina, Utah, and Wisconsin), the geographic area covered and record source type reviewed were the same in 2012 and 2010. Therefore, these eight sites were included in analyses comparing estimated ASD prevalence in 2010 and 2012. For two sites (Arkansas and Maryland), there was a change in geographic area and/or record source type. South Carolina contributed data in 2012 but not in 2010. An ADDM Network site located in Alabama conducted ASD surveillance for part of the 2012 surveillance year, but because of the loss of access to health care data sources, data from Alabama were not complete for the 2012 surveillance year and are not included in this report.

## Results

### Population Characteristics

The geographic surveillance area for the 11 ADDM Network sites in 2012 included 346,978 children aged 8 years, which comprised 8.5% of the U.S. population of children aged 8 years for that year (Table 1). The population distribution of children by race/ethnicity varied by study site. In the pooled data, the population was 53.3% non-Hispanic white, 21.4% non-Hispanic black, 19.8% Hispanic, 4.8% Asian/Pacific Islander, and 0.6% American Indian/Alaska Native.

**TABLE 1:** Number* and percentage of children aged 8 years, by race/ethnicity and site — Autism and Developmental Disabilities Monitoring Network, 11 sites, United States, 2012

Site	Site institution	Surveillance area	Total	White, non-Hispanic		Black, non-Hispanic		Hispanic		API, non-Hispanic		AI/AN, non-Hispanic	
			No.	No.	(%)	No.	(%)	No.	(%)	No.	(%)	No.	(%)
Arizona	University of Arizona	Part of 1 county in metropolitan Phoenix	**32,615**	15,525	(47.6)	1,856	(5.7)	13,180	(40.4)	1,276	(3.9)	778	(2.4)
Arkansas	University of Arkansas for Medical Sciences	16 counties in Arkansas	**14,153**	9,083	(64.2)	3,739	(26.4)	1,025	(7.2)	226	(1.6)	80	(0.6)
Colorado	Colorado Department of Public Health and Environment	7 counties including metropolitan Denver	**40,538**	22,370	(55.2)	2,469	(6.1)	13,448	(33.2)	2,029	(5.0)	222	(0.5)
Georgia	CDC	5 counties including metropolitan Atlanta	**49,720**	16,451	(33.1)	20,556	(41.3)	9,019	(18.1)	3,588	(7.2)	106	(0.2)
Maryland^†^	Johns Hopkins University	1 county in suburban Baltimore	**9,577**	5,019	(52.4)	3,171	(33.1)	656	(6.8)	696	(7.3)	35	(0.4)
Maryland**^§^**	Johns Hopkins University	5 counties in suburban Baltimore	**18,154**	12,293	(67.7)	3,042	(16.8)	1,384	(7.6)	1,383	(7.6)	52	(0.3)
Missouri	Washington University–St. Louis	5 counties including metropolitan St. Louis	**25,870**	17,211	(66.5)	6,516	(25.2)	1,109	(4.3)	970	(3.7)	64	(0.2)
New Jersey	Rutgers University–New Jersey Medical School	4 counties including metropolitan Newark	**32,581**	13,829	(42.4)	7,100	(21.8)	9,787	(30.0)	1,781	(5.5)	84	(0.3)
North Carolina	University of North Carolina–Chapel Hill	11 counties in central North Carolina	**38,913**	20,789	(53.4)	9,544	(24.5)	6,517	(16.7)	1,906	(4.9)	157	(0.4)
South Carolina	Medical University of South Carolina	23 counties in coastal and Pee Dee regions	**24,356**	12,485	(51.3)	9,404	(38.6)	1,964	(8.1)	387	(1.6)	116	(0.5)
Utah	University of Utah	3 counties in northern Utah	**24,945**	18,217	(73.0)	568	(2.3)	4,851	(19.4)	1,151	(4.6)	158	(0.6)
Wisconsin	University of Wisconsin–Madison	10 counties in southeastern Wisconsin	**35,556**	21,758	(61.2)	6,342	(17.8)	5,915	(16.6)	1,392	(3.9)	149	(0.4)
**Total**			**346,978**	**185,030**	**(53.3)**	**74,307**	**(21.4)**	**68,855**	**(19.8)**	**16,785**	**(4.8)**	**2,001**	**(0.6)**

**Abbreviations:** AI/AN = American Indian/Alaska Native; API = Asian/Pacific Islander.

*Total numbers of children aged 8 years in each surveillance area were obtained from CDC’s July 1, 2012 bridged-race population estimates.

^†^Education and health records review area.

^§^Health records only review area.

### Record Review

A total of 48,304 records for 38,038 children aged 8 years were reviewed from education and health care sources. Among these, the source records of 9,629 (19%) children met the criteria for abstraction and subsequently were reviewed by clinicians. Of these 9,629 children, 5,021 (52%) met the ASD surveillance case criteria.

### Birth Certificate Linkage

Of the 5,021 children meeting the ASD surveillance case criteria, 3,848 children (77%) were born in the state where the ADDM Network surveillance site is located, as confirmed by a match to a birth certificate from that state. This percentage ranged from 68% (South Carolina) to 86% (Missouri). The percentage of children who were matched to a birth certificate did not vary by sex or race/ethnicity.

### Overall ASD Prevalence Estimates

**FIGURE 1:**
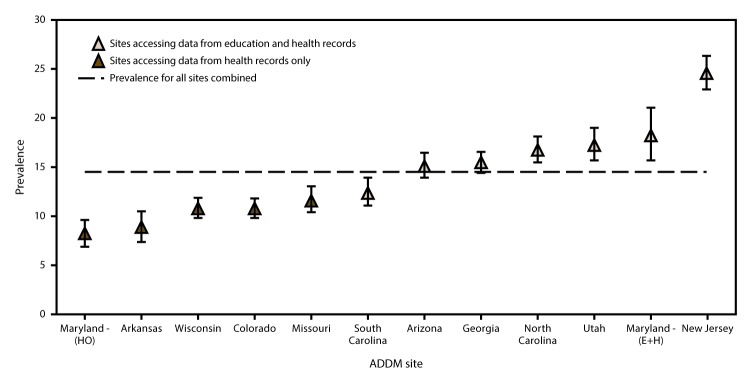
Estimated prevalence* of autism spectrum disorder among children aged 8 years — Autism and Developmental Disabilities Monitoring Network, 11 sites, United States, 2012 **Abbreviations:** ADDM = Autism and Developmental Disabilities Monitoring Network; E+H = education and health records; HO = health records only. *Cases per 1,000 children aged 8 years. Bars represent 95% confidence intervals.

Overall estimated ASD prevalence for the 2012 surveillance year was 14.5 per 1,000 (one in 69) children aged 8 years, on the basis of pooled data from 11 ADDM sites (range: 8.2 [Maryland, health records only reviewed] to 24.6 [in New Jersey]) (Table 2). Estimated ASD prevalence was highest in New Jersey (24.6), Maryland (education and health records reviewed, 18.2), Utah (17.3), and North Carolina (16.8). The seven areas (Arizona, Georgia, Maryland [education and health records reviewed], New Jersey, North Carolina, South Carolina, and Utah) with access to both education and health care sources had higher estimated ASD prevalence compared with the five areas (Arkansas, Colorado, Maryland [health records only reviewed], Missouri, and Wisconsin) with limited or no access to education records (17.1 and 10.4 per 1,000 children aged 8 years, respectively; PR: 1.6; 95% CI: 1.5–1.7; p<0.001) (Figure 1).

**TABLE 2:** Estimated prevalence* of autism spectrum disorder among 1,000 children aged 8 years, by sex — Autism and Developmental Disabilities Monitoring Network, 11 Sites, United States, 2012

Site	Total	Total no. with ASD	Sex			Male-to-female prevalence ratio^†^
			Total^†^	Male	Female	
			Prevalence (95% CI)	Prevalence (95% CI)	Prevalence (95% CI)	
Arizona	**32,615**	**494**	**15.1 (13.9–16.5)**	24.3 (22.0–26.7)	5.7 (4.7–7.0)	4.2 (3.4–5.3)
Arkansas	**14,153**	**125**	**8.8 (7.4–10.5)**	14.1 (11.6–17.1)	3.4 (2.3–5.1)	4.1 (2.6–6.4)
Colorado	**40,538**	**436**	**10.8 (9.8–11.8)**	17.1 (15.4–19.0)	4.2 (3.4–5.2)	4.1 (3.2–5.2)
Georgia	**49,720**	**771**	**15.5 (14.4–16.6)**	25.6 (23.7–27.6)	5.1 (4.3–6.1)	5.0 (4.1–6.0)
Maryland^§^	**9,577**	**174**	**18.2 (15.7–21.1)**	29.4 (25.0–34.6)	6.2 (4.3–9.0)	4.7 (3.2–7.0)
Maryland^¶^	**18,154**	**148**	**8.2 (6.9–9.6)**	13.9 (11.7–16.5)	2.2 (1.4–3.5)	6.2 (3.9–10.0)
Missouri	**25,870**	**300**	**11.6 (10.4–13.0)**	19.0 (16.8–21.5)	3.9 (2.9–5.1)	4.9 (3.6–6.7)
New Jersey	**32,581**	**803**	**24.6 (23.0–26.4)**	39.3 (36.4–42.4)	9.2 (7.8–10.8)	4.3 (3.6–5.1)
North Carolina	**38,913**	**653**	**16.8 (15.5–18.1)**	27.4 (25.1–29.8)	6.0 (5.0–7.2)	4.6 (3.7–5.6)
South Carolina	**24,356**	**302**	**12.4 (11.1–13.9)**	19.9 (17.5–22.5)	4.6 (3.5–6.0)	4.3 (3.2–5.8)
Utah	**24,945**	**431**	**17.3 (15.7–19.0)**	27.7 (24.9–30.7)	6.4 (5.1–8.0)	4.3 (3.4–5.5)
Wisconsin	**35,556**	**384**	**10.8 (9.8–11.9)**	17.2 (15.4–19.2)	4.1 (3.2–5.2)	4.2 (3.2–5.4)
**Total**	**346,978**	**5,021**	**14.5 (14.1–14.9)**	**23.4 (22.7–24.1)**	**5.2 (4.9, 5.6)**	**4.5 (4.2–4.8)**

**Abbreviations:** ASD = autism spectrum disorder; CI = confidence interval.

*Per 1,000 children aged 8 years.

^†^All sites identified significantly higher prevalence among males compared with females (Pearson chi-square, p<0.01).

^§^Education and health records review area.

^¶^Health records only review area.

### Prevalence by Sex and Race/Ethnicity

Across the 11 ADDM Network sites, estimated ASD prevalence among children aged 8 years was 23.4 per 1,000 (one in 43) boys and 5.2 per 1,000 (one in 193) girls; site-specific ASD estimates for boys ranged from 13.9 per 1,000 (in Maryland [health records only reviewed]) to 39.3 per 1,000 (in New Jersey), and for girls from 2.2 per 1,000 (in Maryland) to 9.2 per 1,000 (in New Jersey) (Table 2). The overall prevalence ratio for boys compared with girls was 4.5 (95% CI: 4.2–4.8; p<0.001); site-specific male-to-female prevalence ratios ranged from 4.1 (in Colorado) to 6.2 (in Maryland [health records only reviewed]) and were all statistically significant (Table 2). Estimated prevalence among non-Hispanic white children (15.3 per 1,000) was significantly higher than it was among non-Hispanic black children (13.1 per 1,000; PR: 1.2, 95% CI: 1.1–1.3; p<0.001), Asian/Pacific Islander children (11.4 per 1,000; PR: 1.4, 95% CI: 1.2–1.6; p<0.001), and Hispanic children (10.2 per 1,000; PR: 1.5, 95% CI: 1.4–1.6; p<0.001) (Table 3). Prevalence ratios by sex and race/ethnicity were similar between the areas that reviewed education and health records and the areas that reviewed health records only (Tables 2 and 3).

**TABLE 3:** Estimated prevalence* of autism spectrum disorder among 1,000 children aged 8 years, by race/ethnicity — Autism and Developmental Disabilities Monitoring Network, 11 Sites, United States, 2012

Site	Race/Ethnicity				Prevalence ratio		
	White, non-Hispanic	Black, non-Hispanic	Hispanic	API, non-Hispanic	White-to-black	White-to-Hispanic	Black-to-Hispanic
	Prevalence (95% CI)	Prevalence (95% CI)	Prevalence (95% CI)	Prevalence (95% CI)			
Arizona	16.9 (15.0–19.1)	19.4 (14.0–26.9)	11.3 (9.6–13.3)	13.3 (8.3–21.4)	0.9	1.5^†^	1.7^†^
Arkansas	8.9 (7.2–11.1)	8.0 (5.6–11.5)	—^§^	—	1.1	—	—
Colorado	12.2 (10.9–13.8)	10.5 (7.2–15.5)	6.5 (5.2–8.0)	8.4 (5.2–13.5)	1.2	1.9^†^	1.6^†^
Georgia	18.3 (16.3–20.5)	13.7 (12.2–15.4)	9.0 (7.2–11.2)	13.7 (10.3–18.1)	1.3^†^	2.0^†^	1.5^†^
Maryland^¶^	18.5 (15.1–22.7)	18.6 (14.4–24.0)	12.2 (6.1–24.4)	10.1 (4.8–21.1)	1.0	1.5	1.5
Maryland**	8.6 (7.1–10.4)	6.9 (4.5–10.6)	5.8 (2.9–11.6)	—	1.2	1.5	1.2
Missouri	12.0 (10.5–13.8)	9.1 (7.0–11.7)	8.1 (4.2–15.6)	—	1.3	1.5	1.1
New Jersey	26.8 (24.2–29.7)	23.7 (20.3–27.5)	17.9 (15.4–20.7)	21.9 (16.0–30.0)	1.1	1.5^†^	1.3^†^
North Carolina	18.7 (16.9–20.7)	15.5 (13.2–18.2)	9.1 (7.0–11.7)	18.4 (13.2–25.6)	1.2	2.1^†^	1.7^†^
South Carolina	12.7 (10.8–14.8)	10.6 (8.7–12.9)	6.6 (3.8–11.4)	—	1.2	1.9^†^	1.6
Utah	17.7 (15.8–19.7)	12.3 (5.9–25.9)	13.2 (10.3–16.9)	5.2 (2.3–11.6)	1.4	1.3^†^	0.9
Wisconsin	12.0 (10.6–13.5)	5.8 (4.2–8.1)	7.4 (5.5–10.0)	3.6 (1.5–8.6)	2.1^†^	1.6^†^	0.8
**Total**	**15.3 (14.7–15.8)**	**13.1 (12.3–13.9)**	**10.2 (9.4–10.9)**	**11.4 (9.9–13.1)**	**1.2^†^**	**1.5^†^**	**1.3^†^**

**Abbreviations:** API = Asian/Pacific Islander; CI = confidence interval.

*Per 1,000 children aged 8 years.

^†^Prevalence ratio significant at p<0.05.

^§^Prevalence not calculated when n<5.

^¶^Education and health records review area.

**Health records only review area.

### Intellectual Ability

**FIGURE 2:**
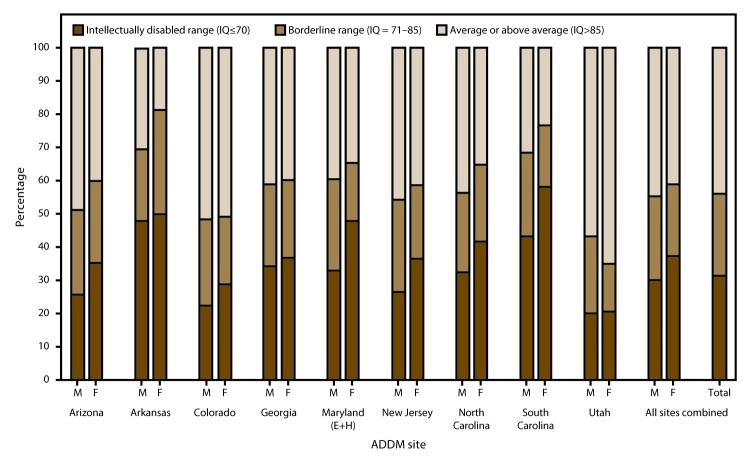
Scores of most recent intelligence quotient tests for children identified with autism spectrum disorder for whom test data were available — Autism and Developmental Disabilities Monitoring Network, nine sites,* United States, 2012 **Abbreviations:** ADDM = Autism and Developmental Disabilities Monitoring Network; ASD = autism spectrum disorder; E+H = education and health records; IQ = intelligence quotient. *Includes sites having information on intellectual ability available for ≥70% of children who met the ASD case definition (N = 3,353, excluding unknown IQ).

Nine ADDM Network areas (Arizona, Arkansas, Colorado, Georgia, Maryland [education and health records review area], New Jersey, North Carolina, South Carolina, and Utah) had data on intellectual ability for at least two thirds of ASD cases (range: 68% [Arkansas] to 92% [in North Carolina]). In these nine areas, 3,353 (80%) of 4,189 children with ASD had data on intellectual ability; for most areas, this percentage did not vary by sex or race/ethnicity, with the exception of Georgia, where the percentage of ASD cases with data on intellectual ability was significantly higher for boys compared with girls (87% and 79%, respectively; p<0.05). Among all 3,353 children, 31.4% were classified in the range of intellectual disability (IQ score ≤70 or the presence of an examiner’s statement of intellectual disability), 24.6% were classified in the borderline range (IQ: 71–85), and 44.1% were classified in the average or above average range (IQ >85 or the presence of an examiner’s statement of average or above average intellectual ability) (Figure 2). The percentage of children classified in the intellectual disability range varied widely across the nine areas, ranging from 20% (in Utah) to 48% (in Arkansas). The percentage of ASD cases classified in the intellectual disability range was significantly higher among girls compared with boys in all nine areas combined (37% and 30%, respectively; p<0.01).

**FIGURE 3:**
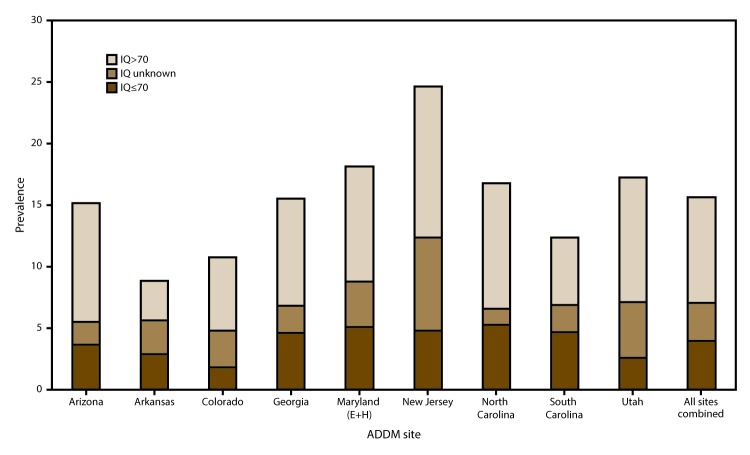
Estimated prevalence* of autism spectrum disorder among children aged 8 years, by most recent intelligence quotient score and by site — Autism and Developmental Disabilities Monitoring Network, nine sites,**^†^** United States, 2012 **Abbreviations:** ADDM = Autism and Developmental Disabilities Monitoring Network; ASD = autism spectrum disorder; E+H = education and health records; IQ = intelligence quotient. *Cases per 1,000 children aged 8 years. ^†^Includes sites having information on intellectual ability available for ≥70% of children who met the ASD case definition (N = 4,189, including unknown IQ). Maryland source type is education and health records.

**FIGURE 4:**
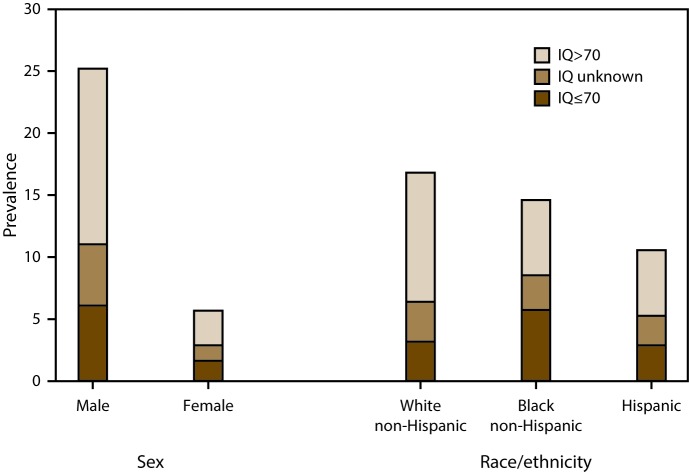
Estimated prevalence* of autism spectrum disorder among children aged 8 years, by most recent intelligence quotient score, by sex and race/ethnicity — Autism and Developmental Disabilities Monitoring Network, nine sites,**^†^** United States, 2012 **Abbreviation:** IQ = intelligence quotient. *Cases per 1,000 children aged 8 years. ^†^Includes nine sites (Arizona, Arkansas, Colorado, Georgia, Maryland [education and health records], New Jersey, North Carolina, South Carolina, and Utah) having information on intellectual ability available for ≥70% of children who met the ASD case definition (N = 4,189, including unknown IQ).

Combining data from all nine sites, the estimated prevalence of ASD with intellectual disability was 3.9 per 1,000 and ranged from 1.8 per 1,000 (in Colorado) to 5.3 per 1,000 (in North Carolina) (Figure 3). The estimated prevalence of ASD without intellectual disability was 8.6 per 1,000 and ranged from 3.2 per 1,000 (in Arkansas) to 12.2 per 1,000 (in New Jersey) (Figure 3). There was a greater male-to-female prevalence ratio for ASD without intellectual disability (PR: 5.1; 95% CI: 4.5–5.7; p<0.001) than for ASD with intellectual disability (PR: 3.7; 95% CI: 3.2–4.3; p<0.001) (Figure 4). The estimated prevalence of ASD with intellectual disability was significantly lower for non-Hispanic white children (3.2 per 1,000) compared with non-Hispanic black children (5.7 per 1,000; PR: 0.6; 95% CI: 0.5–0.6; p<0.001) (Figure 4).

### Early Developmental Concerns and Earliest Comprehensive Evaluation

Analyses of the presence of early developmental concerns and earliest comprehensive evaluation were restricted to children born in the state where the ADDM Network surveillance site was located in order to reduce bias associated with the inability to review early evaluations for children who moved from their state of birth prior to ascertainment by the ADDM Network at age 8 years. Across all ADDM Network sites, 87% of children meeting the ASD surveillance case criteria had documentation of developmental concerns at age ≤36 months in a health or education record (Table 4). This percentage was the same for areas that reviewed education and health records compared with areas that reviewed health records only (87% for each); the percentage was significantly higher for non-Hispanic black children (91%) and for Hispanic children (90%) compared with non-Hispanic white children (86%; p<0.05). The percentage of children with developmental concerns at age ≤36 months was significantly higher for children with ASD and intellectual disability compared with children with ASD without intellectual disability (95% and 85%, respectively; p<0.001).

**TABLE 4:** Number and percentage of children aged 8 years* identified with autism spectrum disorder who received a comprehensive evaluation by a qualified professional at age ≤36 months, 37–48 months, or >48 months, and those with a mention of a developmental concern by age 36 months — Autism and Developmental Disabilities Monitoring Network, 11 sites, United States, 2012

Site	Earliest age when child received a comprehensive evaluation			Mention of a developmental concern by age 36 months
	≤36 mos	37–48 mos	>48 mos	
	No. (%)	No. (%)	No. (%)	No. (%)
Arizona	149 (39.2)	70 (18.4)	161 (42.4)	341 (89.7)
Arkansas	23 (22.8)	32 (31.7)	46 (45.5)	88 (87.1)
Colorado	131 (40.6)	60 (18.6)	132 (40.9)	278 (86.1)
Georgia	222 (41.1)	113 (20.9)	205 (38.0)	473 (87.6)
Maryland^†^	83 (55.0)	27 (17.9)	41 (27.2)	143 (94.7)
Maryland^§^	34 (31.2)	22 (20.2)	53 (48.6)	101 (92.7)
Missouri	103 (40.6)	34 (13.4)	117 (46.1)	210 (82.7)
New Jersey	289 (43.1)	140 (20.9)	241 (36.0)	547 (81.6)
North Carolina	288 (60.1)	71 (14.8)	120 (25.1)	442 (92.3)
South Carolina	79 (38.5)	52 (25.4)	74 (36.1)	189 (92.2)
Utah	120 (37.7)	66 (20.8)	132 (41.5)	259 (81.4)
Wisconsin	133 (41.8)	63 (19.8)	122 (38.4)	286 (89.9)
**Total**	**1,654 (43.0)**	**750 (19.5)**	**1,444 (37.5)**	**3,357 (87.2)**

*Includes 3,848 children identified with autism spectrum disorder who were linked to an in-state birth certificate.

^†^Education and health records review area.

^§^Health records only review area.

Using combined data from all sites for children meeting the ASD surveillance case criteria and restricting to children born in the state where the ADDM Network surveillance site was located, the earliest known comprehensive evaluation occurred at age ≤36 months for 43% of children, between 37 and 48 months for 20% of children, and after 48 months for the remaining 38% of children (Table 4). This percentage did not vary between boys and girls (42% and 46%, respectively; p>0.05), but was significantly higher for non-Hispanic white children (45%) compared with non-Hispanic black children (40%; p<0.05) and with Hispanic children (39%; p<0.05) (data not shown). Children with ASD and intellectual disability were more likely to have an earliest known comprehensive evaluation by age 36 months compared with children with ASD without intellectual disability (55% and 38%, respectively; p<0.001) (data not shown). The median age at earliest known comprehensive evaluation was 40 months, ranging from 30 months (North Carolina) to 48 months (Arkansas) (data not shown).

### Earliest Known ASD Diagnosis and Diagnosis Category

On the basis of pooled data from all ADDM Network sites, 74% of children identified with ASD had an earliest known DSM-IV-TR ASD diagnosis of autistic disorder (46%), ASD-NOS/PDD-NOS (44%), or Asperger disorder (10%) given by a community provider (Table 5). The median age at the earliest known diagnosis was 50 months overall and was lower for autistic disorder (46 months) compared with ASD-NOS/PDD-NOS (49 months; p<0.01) and with Asperger disorder (74 months; p<0.001) (Table 5). Within each specific diagnosis subtype, there were no differences in median age at earliest known diagnosis by sex or race/ethnicity (data not shown).

**TABLE 5:** Median age of earliest known autism spectrum disorder diagnosis and number and proportion within each diagnostic subtype — Autism and Developmental Disabilities Monitoring Network, 11 sites, United States, 2012

Site	ASD subtype							
	Autistic disorder		ASD-NOS/PDD-NOS		Asperger disorder		Any ASD subtype	
	Median age (mos)	No. (%)	Median age (mos)	No. (%)	Median age (mos)	No. (%)	Median age (mos)	No. (%)
Arizona	50.0	253 (74.4)	64.0	72 (21.2)	77.0	15 (4.4)	55.0	340 (68.8)
Arkansas	57.0	73 (76.0)	60.0	12 (12.5)	84.0	11 (11.5)	60.0	96 (76.8)
Colorado	48.0	184 (66.2)	59.0	55 (19.8)	80.0	39 (14.0)	55.0	278 (63.8)
Georgia	47.5	261 (48.0)	51.0	230 (42.2)	71.0	53 (9.7)	51.0	544 (70.6)
Maryland*	41.0	56 (40.6)	48.0	79 (57.2)	44.0	3 (2.2)	45.5	138 (79.3)
Maryland^†^	46.5	44 (35.5)	44.5	72 (58.1)	74.0	8 (6.5)	48.0	124 (83.8)
Missouri	50.0	67 (26.0)	51.0	145 (56.2)	77.5	46 (17.8)	58.0	258 (86.0)
New Jersey	45.0	193 (29.7)	43.0	380 (58.5)	74.0	77 (11.8)	47.0	650 (80.9)
North Carolina	36.5	206 (53.6)	55.0	155 (40.4)	72.0	23 (6.0)	48.0	384 (58.8)
South Carolina	45.0	143 (65.6)	58.5	68 (31.2)	74.0	7 (3.2)	48.0	218 (72.2)
Utah	45.0	114 (31.7)	48.0	178 (49.4)	63.5	68 (18.9)	50.0	360 (83.5)
Wisconsin	45.5	106 (34.8)	49.0	173 (56.7)	74.0	26 (8.5)	50.0	305 (79.4)
**Total**	**46.0**	**1,700 (46.0)**	**49.0**	**1,619 (43.8)**	**74.0**	**376 (10.2)**	**50.0**	**3,695 (73.6)**

**Abbreviations:** ASD = autism spectrum disorder; ASD-NOS = autism spectrum disorder–not otherwise specified; PDD-NOS = pervasive developmental disorder–not otherwise specified.

*Education and health records review area.

^†^Health records only review area.

### Special Education Eligibility

The seven ADDM Network areas that reviewed records at education sources obtained data on the eligibility categories through which children with ASD were served in the public school special education system. Combined data from these seven areas indicate that 79% of children with ASD had special education records; this percentage ranged from 55% (Utah) to 92% (Arizona). Among these children, more than half had a primary special education eligibility classification of autism (range: 53% [in Utah]–70% [in Maryland education and health records review area]) (Table 6). Combining data from all seven areas, the percentage of children with an autism eligibility classification did not vary between boys (61%) and girls (57%; p>0.05) or between non-Hispanic white (58%) and Hispanic children (56%; p>0.05) but was greater for non-Hispanic black children (64%) compared with non-Hispanic white (p<0.01) and Hispanic (p<0.01) children (data not shown).

**TABLE 6:** Number and percentage of children aged 8 years identified with autism spectrum disorder with available special education records, by primary special education eligibility category* — Autism and Developmental Disabilities Monitoring Network, seven sites with access to education records, United States, 2012

Primary special education eligibility category	Arizona	Georgia	Maryland^†^	New Jersey	North Carolina	South Carolina	Utah
Autism (%)	61.4	58.1	70.2	56.1	69.1	61.0	53.4
Emotional disturbance (%)	4.6	1.1	1.6	0.7	1.4	0	1.7
Specific learning disability (%)	6.8	3.1	8.1	4.6	7.9	4.9	8.5
Speech or language impairment (%)	6.8	1.6	0	10.4	2.8	2.7	18.6
Hearing or visual impairment (%)	0	0.6	0	0.3	0	0	0
Health or physical disability (%)	4.4	3.7	12.1	19.2	10.3	5.4	10.6
Multiple disabilities (%)	2.0	0	4.0	6.4	0.8	0	0
Intellectual disability (%)	8.1	2.6	3.2	1.0	3.8	3.6	4.2
Developmental delay/preschool (%)	5.7	29.1	0.8	0.4	3.8	20.2	3.0
Other (%)	0	0	0	0.9	0.2	2.2	0
**Total no. of ASD cases**	**494**	**771**	**174**	**803**	**653**	**302**	**431**
**Total no. (%) of ASD cases with special education records**	**454 (91.9)**	**621 (80.5)**	**124 (71.3)**	**702 (87.4)**	**505 (77.3)**	**223 (73.8)**	**236 (54.8)**

**Abbreviation:** ASD = autism spectrum disorder.

*Some state-specific categories were recoded or combined to match current US Department of Education categories.

^†^Education and health records review area.

### Previously Documented ASD Classification

**FIGURE 5:**
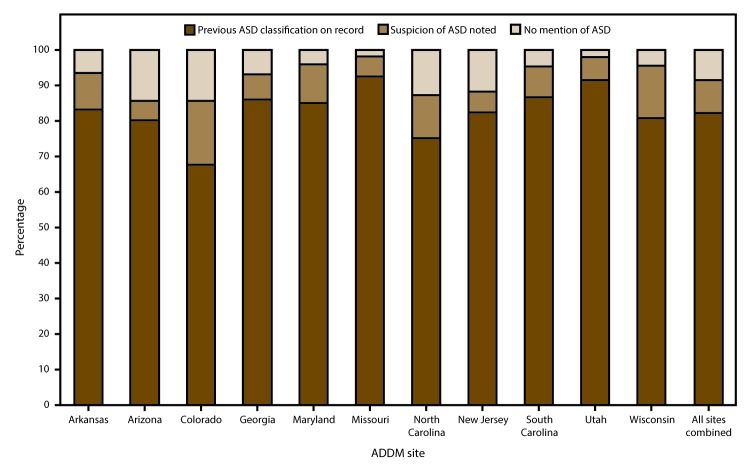
Percentage of children with autism spectrum disorder at age 8 years who had previous autism spectrum disorder classification on record, suspicion of the disorder noted, or no mention of the disorder, by site — Autism and Developmental Disabilities Monitoring Network, 11 sites, United States, 2012 **Abbreviations:** ADDM = Autism and Developmental Disabilities Monitoring Network; ASD = autism spectrum disorder.

Across the 11 ADDM Network sites, 82% of children who met the ASD surveillance case criteria had either a previous diagnosis of ASD or a documented eligibility classification of autism in the special education system, 9% had a suspicion of ASD documented in an evaluation, and the remaining 9% had no mention of ASD in the records (Figure 5). At individual ADDM Network sites, the percentage of children with a previous ASD diagnosis or eligibility classification ranged from 68% (Colorado) to 93% (Missouri) (Figure 5). The percentage of children with a previous ASD diagnosis or eligibility classification was the same for boys and girls (82%) and similar for non-Hispanic white and non-Hispanic black children (82% and 84%, respectively), but was significantly lower for Hispanic children (78%) compared with non-Hispanic white children (p<0.01) and non-Hispanic black children (p<0.001) (data not shown).

### Comparison of ASD Prevalence Estimates Between 2010 and 2012

For eight sites (Arizona, Colorado, Georgia, Missouri, New Jersey, North Carolina, Utah, and Wisconsin), the geographic areas covered and record source types reviewed were the same for 2010 and 2012. On the basis of combined data from these eight sites in each respective year, estimated ASD prevalence was 15.1 and 15.2 per 1,000 children aged 8 years in 2010 and 2012, respectively (p>0.05) (Table 7). Estimated ASD prevalence for male, female, non-Hispanic white, non-Hispanic black, or Hispanic children did not differ significantly between 2010 and 2012. Five of these eight sites collected data on intellectual ability for at least two thirds of the children identified with ASD. On the basis of combined data from these five sites in each respective year, estimated ASD prevalence was 17.5 and 17.6 per 1,000 children aged 8 years in 2010 and 2012, respectively (p>0.05) and was similar between 2010 and 2012 for male, female, non-Hispanic white, non-Hispanic black, and Hispanic children (Table 8). Prevalence estimates were similar for 2010 and 2012 for children with ASD with intellectual disability and for children with ASD without intellectual disability.

**TABLE 7:** Comparison of autism spectrum disorder prevalence among sites with comparable surveillance areas in 2010 and 2012, by record source type, sex, and race/ethnicity, Autism and Developmental Disabilities Monitoring Network, eight sites, United States

Characteristic	2010	2012	2012-to-2010 prevalence ratio (95% CI)
	Prevalence (95% CI)	Prevalence (95% CI)	
**Record source**			
E+H areas*	17.5 (16.9–18.2)	17.6 (17.0–18.3)	1.00 (0.95–1.05)
HO areas^†^	10.8 (10.1–11.4)	11.0 (10.3–11.7)	1.02 (0.94–1.11)
E+H-to-HO prevalence ratio	1.6 (1.5–1.7)	1.6 (1.5–1.7)	—^§^
**Site**			
Arizona	15.7 (14.4–17.1)	15.1 (13.9–16.5)	0.97 (0.85–1.09)
Colorado	9.9 (9.0–10.9)	10.8 (9.8–11.8)	1.09 (0.95–1.25)
Georgia	15.5 (14.5–16.7)	15.5 (14.5–16.6)	1.00 (0.90–1.10)
Missouri	14.2 (12.8–15.7)	11.6 (10.4–13.0)	0.82 (0.70–0.96)
New Jersey	21.9 (20.4–23.6)	24.6 (23.0–26.4)	1.11 (1.01–1.23)
North Carolina	17.3 (16.1–18.7)	16.8 (15.5–18.1)	0.97 (0.87–1.08)
Utah	18.6 (17.0–20.4)	17.3 (15.7–19.0)	0.93 (0.81–1.06)
Wisconsin	9.3 (8.3–10.3)	10.8 (9.8–11.9)	1.17 (1.01–1.35)
**Sex**			
Male	24.4 (23.6–25.2)	24.6 (23.8–25.4)	1.01 (0.96–1.05)
Female	5.4 (5.0–5.8)	5.5 (5.1–5.9)	1.02 (0.92–1.13)
Male-to-female prevalence ratio	4.5 (4.2–4.9)	4.5 (4.1–4.8)	—
**Race/Ethnicity**			
White, non-Hispanic	16.2 (15.5–16.8)	16.5 (15.8–17.2)	1.02 (0.96–1.08)
Black, non-Hispanic	12.9 (12.0–13.9)	13.9 (12.9–14.9)	1.08 (0.97–1.19)
Hispanic	11.2 (10.4–12.1)	10.5 (9.7–11.3)	0.93 (0.84–1.04)
White-to-black prevalence ratio	1.3 (1.2–1.4)	1.2 (1.1–1.3)	—
White-to-Hispanic prevalence ratio	1.4 (1.3–1.6)	1.6 (1.4–1.7)	—
Black-to-Hispanic prevalence ratio	1.2 (1.0–1.3)	1.3 (1.2–1.5)	—
**Total**	**15.1 (14.6–15.5)**	**15.2 (14.8**–**15.7)**	**1.01 (0.97**–**1.05)**

**Abbreviations:** CI = confidence interval; E+H = education and health records review; HO = health records only review.

*Sites reviewing education and health records: Arizona, Georgia, New Jersey, North Carolina, and Utah.

^†^Sites reviewing health records only: Colorado, Missouri, and Wisconsin.

^§^Ratios of prevalence ratios were not calculated.

**TABLE 8:** Comparison of autism spectrum disorder prevalence among sites with comparable surveillance areas, by sex, race/ethnicity, and most recent score on intelligence quotient test, Autism and Developmental Disabilities Monitoring Network, five sites,* United States, 2010 and 2012

Characteristic	2010	2012	Prevalence ratio 2012-to-2010 (95% CI)
	Prevalence (95% CI)	Prevalence (95% CI)	
**Sex**			
Male	28.5 (27.4–29.6)	28.5 (27.5–29.7)	1.00 (0.95–1.06)
Female	6.2 (5.7–6.8)	6.3 (5.8–6.9)	1.02 (0.91–1.15)
Male-to-female prevalence ratio	4.6 (4.2–5.0)	4.5 (4.1–4.9)	—^†^
**Race/Ethnicity**			
White, non-Hispanic	19.4 (18.5–20.4)	19.4 (18.5–20.4)	1.00 (0.93–1.07)
Black, non-Hispanic	15.2 (14.0–16.4)	16.2 (15.0–17.5)	1.07 (0.95–1.19)
Hispanic	13.5 (12.4–14.6)	12.2 (11.2–13.3)	0.90 (0.80–1.02)
White-to-black prevalence ratio	1.3 (1.2–1.4)	1.2 (1.1–1.3)	—
White-to-Hispanic prevalence ratio	1.5 (1.3–1.6)	1.6 (1.4–1.8)	—
Black-to-Hispanic prevalence ratio	1.1 (1.0–1.3)	1.3 (1.2–1.5)	—
**IQ**			
≤70	4.6 (4.3–4.9)	4.3 (4.0–4.7)	0.94 (0.85–1.04)
>70	10.6 (10.1–11.1)	10.0 (9.6–10.5)	0.95 (0.89–1.01)
Unknown	2.4 (2.1–2.6)	3.3 (3.0–3.6)	1.39 (1.23–1.58)
>70-to-≤70 prevalence ratio	2.3 (2.1–2.5)	2.3 (2.1–2.5)	—
**Total**	**17.5 (16.9–18.2)**	17.6 (17.1–18.3)	1.01 (0.96–1.06)

**Abbreviations**: CI = confidence interval; IQ = intelligence quotient.

*Arizona, Georgia, New Jersey, North Carolina, and Utah.

^†^Ratios of prevalence ratios were not calculated.

Although overall estimated ASD prevalence between 2010 and 2012 was similar across the eight sites that were comparable between these 2 years, the same was not true of all of the individual surveillance sites, three of which had significantly different prevalence estimates in 2012 compared with 2010. Between these two surveillance years, ASD prevalence increased by 17% in Wisconsin and by 11% in New Jersey and decreased by 18% in Missouri.

### Evaluation of the Effect of Missing Records

An evaluation of the effect of missing records suggested that estimated ASD prevalence might have increased by 0.1% (in Wisconsin) to 3.3% (in Utah) if missing records had been available for review. Across all 11 sites, estimated ASD prevalence might have increased by <1% in four sites (Arizona, Colorado, Missouri, and Wisconsin), by 1%–<2% in three sites (Georgia, North Carolina, and New Jersey) and by 2.0%–3.3% in four sites (Arkansas, Maryland, South Carolina, and Utah).

## Discussion

Estimated ASD prevalence among children aged 8 years in the ADDM Network in 2012 was 14.5 per 1,000, or one in 69. Estimated prevalence was four and a half times higher among boys than among girls; estimated ASD prevalence was 23.4 per 1,000 boys (one in 43 boys) and 5.2 per 1,000 girls (one in 193 girls). Prevalence estimates varied widely among the 11 ADDM Network sites, ranging from 8.2 per 1,000 children aged 8 years (in the area of the Maryland site where health records only were reviewed) to 24.6 per 1,000 (in New Jersey, where both education and health records were reviewed). The estimated prevalence of ASD with intellectual disability was 3.9 per 1,000 overall and ranged from 1.8 per 1,000 (in Colorado) to 5.3 per 1,000 (in North Carolina). The estimated prevalence of ASD without intellectual disability was 8.6 per 1,000 overall, ranged from 3.2 per 1,000 (in Arkansas) to 12.2 per 1,000 (in New Jersey), and exceeded the estimated prevalence of ASD with intellectual disability in all sites. Across all ADDM Network sites, estimated ASD prevalence was 20% higher among non-Hispanic white compared with non-Hispanic black children, 40% higher among non-Hispanic white compared with Asian/Pacific Islander children, 50% higher among non-Hispanic white compared with Hispanic children, and 30% higher among non-Hispanic black compared with Hispanic children.

The overall prevalence estimate for 2012 was nearly identical to the reported estimate for the ADDM Network in 2010 of 14.7 per 1,000, or one in 68 children aged 8 years. However, because of differences between 2010 and 2012 in the geographic area covered and record source types reviewed for some individual ADDM Network sites, comparing the overall prevalence estimates might be misleading. For this reason, comparisons of ASD prevalence estimates between 2010 and 2012 were restricted to the eight sites for which the geographic surveillance area and record source type reviewed were comparable between the two surveillance years, including five sites with sufficient information on intellectual ability among children with ASD for both years. When results were restricted to these eight sites, combined ASD prevalence estimates were similar for 2010 and 2012, including in subgroups defined by sex and race/ethnicity. In the five sites with data on intellectual ability, the estimated prevalence of ASD with and without intellectual disability was unchanged in 2012 compared with 2010. This is notable given that the increase in estimated ASD prevalence that has occurred since 2002 has been accompanied by a greater increase in ASD without intellectual disability than ASD with intellectual disability.

Despite the similar findings when the population was restricted to these eight sites for 2010 and 2012 (15.1 and 15.2 per 1,000, respectively), there were significant differences in ASD prevalence estimates between 2010 and 2012 for three of these sites. Significantly increased ASD prevalence estimates were observed in New Jersey (11%) and Wisconsin (17%). In Missouri, estimated ASD prevalence decreased significantly, by 18%, and at the remaining five sites (Arizona, Colorado, Georgia, North Carolina, and Utah), ASD prevalence estimates did not change. The factors underlying the prevalence estimate changes at individual ADDM Network sites are not clear. The two sites with the greatest change from 2010 to 2012 (Missouri and Wisconsin) both reviewed only health source type records for 2010 and 2012. The ability to obtain a comprehensive developmental evaluation through the health care system might be subject to greater local variation compared with evaluations performed through the education system because of changes in the number and availability of providers, changes in insurance coverage policies, or other factors. In addition, changes in record retention associated with migration to electronic health records could limit the availability of historical evaluations at some sources. The wide range of ASD prevalence estimates reported by sites participating in the 2012 ADDM Network coupled with the prevalence estimate increases at some sites suggest the need for caution in interpreting the similarity of overall estimated ASD prevalence between 2010 and 2012. Data from additional surveillance years are needed to understand the trajectory of ASD prevalence.

Population-based estimates of ASD prevalence in the United States also are reported by two CDC surveys. The National Health Interview Study (NHIS) is a nationally representative household survey, and the National Survey of Children’s Health (NSCH) is a nationally representative survey of households with children aged 0–17 years in the United States. Both surveys base ASD prevalence estimates on parent or caregiver report of being told by a doctor or other health care provider that the child had ASD. Both NHIS and NSCH ask if the parent/caregiver was ever told that the child has ASD (“ever ASD”); NSCH also includes a follow-up question asking whether the child currently has ASD (“current ASD”). A previous analysis showed that 13% of parents who reported ever being told that the child had ASD also reported that the child did not currently have ASD; most of these parents attributed the lack of a current ASD diagnosis to new information, suggesting that basing prevalence estimates on ever ASD might overestimate prevalence compared with current ASD (*25*). For the 2014 NHIS, the prevalence of parent or caregiver-reported ever ASD was 22.4 per 1,000 children aged 3–17 years (*26*). For the 2011–2012 NSCH, the prevalence of parent or caregiver-reported current ASD was 20 per 1,000 children aged 6–17 years (*11*). The 2012 ADDM Network overall ASD prevalence estimate of 14.5 per 1,000 is lower than the overall estimates reported in these surveys; however, differences in the sample population and methodology should be taken into account when comparing results for these three studies. The 2011–2012 NSCH included children aged 6–17 years; when further stratified by age, ASD prevalence was 18.2 per 1,000 children aged 6–9 years and 23.9 per 1,000 children aged 10–13 years. Although the difference in ASD prevalence between these two age groups in the NSCH was not statistically significant, the estimate for children aged 6–9 years (18.2 per 1,000) is closer to the 2012 ADDM Network overall ASD prevalence estimate for children aged 8 years (14.5 per 1,000) and similar to the estimate for the 2012 ADDM Network sites that reviewed education and health care records (17.1 per 1,000). The ASD prevalence estimate from the 2007 NSCH (11.6 per 1,000 children aged 6–17 years) (*13*) was similar to 2008 ADDM Network prevalence estimate (11.3 per 1,000 children aged 8 years) (*17*). Taken as a whole, studies using different methodologies and in different populations have reported converging estimates for ASD prevalence in the United States. Future studies by the ADDM Network will incorporate DSM-5 diagnostic criteria, and ongoing ADDM Network surveillance will provide information regarding ASD prevalence trends using DSM-IV-TR and DSM-5 diagnostic criteria.

Consistent with previous years of ADDM Network surveillance (*16**–**20*), the overall male-to-female ASD prevalence ratio was 4.5 in 2012 and has remained largely unchanged across recent surveillance years: 4.5 in 2004 (*18*), 2006 (*18*), and 2010 (*16*) and 4.6 in 2008 (*17*). A similar male-to-female ASD prevalence ratio was found among school-age children in data from the 2010–2011 NSCH (*11*). Observed differences in estimated ASD prevalence by child characteristics such as sex and race/ethnicity might indicate areas where ASD identification is incomplete and can provide data to inform policies and efforts to improve identification of ASD among subgroups, particularly female and nonwhite children who have historically had lower identified prevalence compared with male and non-Hispanic white children. The higher estimated prevalence among boys might result from sex-specific differences in ASD risk (*27*,*28*) or differences in identification of girls with ASD arising from less well-recognized symptom profiles (*29*), or both. The lower male-to-female prevalence ratio for ASD with intellectual disability (PR: 3.7) compared with ASD without intellectual disability (PR: 5.1) is consistent with data from previous ADDM Network surveillance years. Continued attention should be paid to ensuring that all children with ASD are identified, regardless of functional status.

Results from ADDM Network ASD surveillance in 2012 continue to indicate disparities in estimated ASD prevalence by race/ethnicity. Across all sites, estimated ASD prevalence among non-Hispanic white children was 20% higher compared with non-Hispanic black children, 40% higher compared with Asian/Pacific Islander children, and 50% higher compared with Hispanic children. In addition, a lower percentage of non-Hispanic black and Hispanic children had an earliest comprehensive evaluation by age 36 months compared with non-Hispanic white children. Observed prevalence differences by race/ethnicity might reflect differences in awareness of ASD or access to specialty diagnostic services (*30*). For the Hispanic population, studies have identified lack of awareness of ASD, stigma associated with disability, lack of access to health care services due to noncitizenship or low income, and language barriers as factors that might reduce the identification of ASD among Hispanic children (*31*–*35*). In the 2009–2010 National Survey of Children with Special Health Care Needs (NSCSHCN), estimated ASD prevalence was nearly 50% higher for non-Hispanic white children (15.3 per 1,000) compared with non-Hispanic black children (10.4 per 1,000) and nearly 300% higher for non-Hispanic white children compared with Hispanic children living in households where the primary language was not English (5.2 per 1,000). In contrast, estimated ASD prevalence was similar for non-Hispanic white children compared with Hispanic children living in households where the primary language was English (14.3 per 1,000) (*32*). Language differences could affect the administration and interpretation of developmental screening and monitoring, impede communication of parental concerns about a child’s development or a health care provider’s recommendation for further evaluation, and limit access to programs and campaigns aimed at increasing awareness of ASD. If lower prevalence in non-Hispanic black and Hispanic children indicates that not all non-Hispanic black and Hispanic children with ASD are being evaluated and/or diagnosed in the community, the children who are not identified might not receive ASD-related services and supports, including school supports to facilitate educational progress. Targeted strategies are needed to increase awareness and identification of ASD in minority communities.

The consistently greater ASD prevalence estimated with data from sites that reviewed education and health source type records underscores the role that public schools play in the equitable provision of comprehensive evaluations to children with developmental concerns. The Individuals with Disabilities Education Act mandates that states and school districts identify, locate, and evaluate all children with disabilities at no cost to the family, so comprehensive evaluations provided through school systems might be more accessible and affordable compared with evaluations performed through the health care system. However, results from these evaluations might not be reported to the health care provider or included in the health care provider records. Parents and caregivers should be encouraged to share the results of comprehensive evaluations performed through the school system with the child’s health care provider to improve continuity of care and ensure that the health care provider can make recommendations that are based on the child’s needs.

The early identification of ASD is a priority of the American Academy of Pediatrics, which recommends universal ASD screening at ages 18 and 24 months, and by the U.S. Department of Health and Human Services through the *Healthy People 2020* goal of a 10% increase in the percentage of children with ASD who receive their first evaluation by age 36 months (*22*). ADDM Network data are used to measure the goal that 47% of children with ASD have a first evaluation by age 36 months; the baseline percentage for this goal is 42.7%, as measured by ADDM Network data in 2006. Lowering the age at first evaluation is important because when impairments are identified through a comprehensive evaluation, referrals for specific services can be made, often without a formal diagnosis. On the basis of evidence linking early treatment to improved outcomes (*36*–*39*), it is important that children with developmental concerns be evaluated and referred to services as soon as possible. In 2010, the percentage of children aged 8 years with ASD residing the ADDM catchment area with an earliest known comprehensive evaluation by age 36 months was 43.8% (*16*), and the 2012 percentage was similar at 43%. Although several years remain before determination of whether the goal was achieved, the lack of progress from the baseline measured in 2006 through 2012 is disappointing. Of note, the age cohort represented here was born in 2004 and therefore the findings regarding the percentage of children with an earliest known evaluation by age 36 months reflect practices during 2004–2007. Continued surveillance is necessary to monitor progress towards the *Healthy People 2020* goal, particularly in light of the 2006 AAP screening recommendations, and to identify factors associated with later age at first evaluation so that strategies to improve early referral and evaluation can be developed. ADDM Network surveillance of ASD prevalence and characteristics among children aged 4 years, which began in 2010, can help to provide more timely data on early identification of children with ASD (*40*).

The availability of records containing developmental evaluations conducted to determine eligibility for special education services as well as those conducted through the health care system in response to concerns about a child’s development forms the basis for the public health surveillance of ASD conducted by the ADDM Network. By screening existing records then applying a consistent methodology by trained and research-reliable clinician reviewers to determine case status, the ADDM Network is able to conduct population-based surveillance of ASD in a large and diverse population. This methodology was validated, compared with direct examination of children, and the methods were found to result in a prevalence estimate that is likely conservative (*41*).

## Limitations

The findings in this report are subject to at least seven limitations. First, data were limited to the information available in the source records. The amount and quality of the data define the potential to determine whether a child meets the ASD surveillance case definition and the extent to which the characteristics of the identified population can be described. In particular, data on intellectual ability were not available for all children, and the distribution of intellectual ability among the children with these data might not be generalizable to all children with ASD. Second, the types of source records varied across sites, and the inability to review education records at some sites might have led to an underestimate of ASD prevalence in those sites. Third, education records generally were not available for children attending private school or being home-schooled. Fourth, the surveillance areas were selected through a competitive process and were not selected to be representative of children aged 8 years in the United States or the state where the surveillance site was located. Fifth, county-level population counts for children by sex and race/ethnicity are not available by single year of age in nondecennial census years. Population estimates published by the National Center for Health Statistics are used instead. There is evidence that the error in population estimates for the intervening years between decennial census counts increases with increasing years beyond the decennial census (in this case, 2010) (*42*). Sixth, the analysis of age at first comprehensive evaluation was restricted to children for whom linkage was made to birth certificates for the state where the ADDM Network site was located in an attempt to reduce bias resulting from the unavailability of early evaluations for children who moved after birth. However, a child might have moved out and back into this state between birth and ascertainment, so this restriction might not have completely eliminated this potential source of bias. Finally, race and ethnicity were defined broadly for this surveillance population, and results for a specific race or ethnic group might not be representative of results for all children in these groups. In addition, it was not possible to distinguish Hispanic children living in households in which the primary language was English from those with a different primary language.

## Future Study Directions

In 2013, revised diagnostic criteria for ASD were published by the American Psychiatric Association in the DSM-5 (*5*). Beginning with the 2014 surveillance year, the ADDM Network will be able to estimate ASD case status on the basis of both DSM-5 and DSM-IV-TR. This evaluation is possible because of the data collection methods employed since the inception of the ADDM Network, including the abstraction of specific behaviors documented in children’s records. This unique component of ADDM Network ASD surveillance will enable the ADDM Network investigators to evaluate the change in estimated ASD prevalence that might arise from the change in diagnostic criteria. Previous analyses have suggested that fewer children will meet the behavioral criteria of DSM-5 compared with DSM-IV-TR (*43*). However, DSM-5 criteria include a provision that children with a well-established diagnosis of one of the three autism spectrum disorder subtypes under DSM-IV-TR criteria are considered to have ASD under DSM-5 criteria. Therefore, at least for the initial years following the publication of DSM-5, ASD prevalence estimates that are based on DSM-5 criteria should include the children with a DSM-IV-TR-based diagnosis in order to accurately represent the number of children who are being treated and served for ASD by community providers. Because the surveillance methodology of the ADDM Network also includes collection of information on ASD diagnoses by community providers, future estimates of the prevalence of ASD under DSM-5 will be able to include children who meet DSM-5 criteria by virtue of a past DSM-IV-TR diagnosis as well as those meeting the DSM-5 behavioral criteria.

## Conclusion

Approximately one in 69 children aged 8 years living in sites participating in the ADDM Network surveillance areas met the ASD case criteria for the 2012 surveillance year. Although the overall prevalence estimate is virtually unchanged from surveillance year 2010, prevalence ranged widely across the ADDM Network and prevalence increases were reported at two sites, suggesting that it is premature to conclude that the rising prevalence of ASD observed during the first decade of the 21st century might be slowing. Ongoing surveillance of ASD prevalence through the ADDM Network is likely to provide the most accurate means to monitor trends in ASD prevalence over time, including those that are related to changes in the diagnostic criteria for ASD. ASD surveillance informs providers, particularly public schools, of upcoming service needs, and provides feedback on progress made toward early identification goals. The ADDM Network will continue to track age at first comprehensive evaluation to monitor progress toward the *Healthy People 2020* goal of increasing the percentage of children with ASD who receive a first evaluation by age 36 months. Estimated ASD prevalence was substantially lower among Hispanic and non-Hispanic black children compared with non-Hispanic white children. In addition, non-Hispanic black and Hispanic children were less likely to have a first evaluation by age 36 months and Hispanic children were less likely to have a previous ASD diagnosis or classification. This finding suggests that a number of nonwhite children with ASD are not being identified and evaluated, and for those children who are evaluated, a later age at the first comprehensive evaluation likely delays initiation of services for these children. No intervention has been shown to reduce the prevalence of ASD; however, early treatment might maximize the ability of children to function and participate in their community. Initiation of school-based services prior to formal school entry might help to facilitate optimal educational progress. Continued efforts should be made to promote early identification of all children with ASD so that interventions can be initiated at the youngest age possible.
